# Managing Gestational Diabetes Complexity with Continuous Glucose Monitoring: A Narrative Review

**DOI:** 10.3390/diagnostics16142145

**Published:** 2026-07-08

**Authors:** Anca-Elena Crăciun, Dana Mihaela Ciobanu, Georgeta Inceu, Camelia Larisa Vonica, Denisa Herman, Cristian-Ioan Crăciun, Adriana Fodor, Cornelia Bala, Adriana Rusu

**Affiliations:** 1 Department of Medical Sciences, Faculty of Nursing and Health Sciences, “Iuliu Hațieganu” University of Medicine and Pharmacy, 400006 Cluj-Napoca, Romania; anca.craciun@umfcluj.ro (A.-E.C.); adriana.rusu@umfcluj.ro (A.R.); 2 Diabetes Center, Emergency Clinical County Hospital Cluj, 400006 Cluj-Napoca, Romania; inceu.victoria@umfcluj.ro (G.I.); camelia.sulea@umfcluj.ro (C.L.V.); hermandenisa@yahoo.com (D.H.); adriana.fodor@umfcluj.ro (A.F.); cbala@umfcluj.ro (C.B.); 3 Department of Diabetes and Nutrition Diseases, Faculty of Medicine, “Iuliu Hațieganu” University of Medicine and Pharmacy, 400006 Cluj-Napoca, Romania; 4 Department of Pharmacology, Toxicology and Clinical Pharmacology, “Iuliu Hațieganu” University of Medicine and Pharmacy, 400337 Cluj-Napoca, Romania; doctor.craciun@yahoo.com

**Keywords:** gestational diabetes, continuous glucose monitoring, pregnancy outcome, narrative review

## Abstract

Gestational diabetes (GDM) is a frequent health problem associated with both short- and long-term adverse outcomes for mother and child. Standard management includes lifestyle interventions and, when necessary, pharmacologic therapy. However, the effectiveness and timely initiation of pharmacological therapy depend on accurate glucose monitoring. Continuous glucose monitoring (CGM) systems have emerged as valuable tools in diabetes care, providing real-time information on glycemic variability and enabling more individualized therapeutic interventions. In this narrative review, we explore the role of CGM in the early detection of dysglycemia, its diagnostic and prognostic value, and its ability to identify specific glycemic patterns during pregnancies complicated by GDM. We also assess its role in optimizing lifestyle interventions and guiding pharmacotherapeutic strategies. Current evidence suggests that CGM supports clinical decision-making and patient engaging by providing real-time glucose data. This facilitates earlier identification of hyperglycemic patterns, more precise treatment changes and improved glucose control. Furthermore, CGM use has been associated with improved neonatal and maternal outcomes. Despite these promising findings, barriers such as cost and limited access persist. Although the existing evidence remains relatively limited, it supports the integration of CGM into routine care of women with GDM as part of a comprehensive and personalized treatment strategy. Larger clinical trials are needed to fully understand the benefits and optimal use of CGM in GDM, as well as its impact on pregnancy outcomes, glycemic control and psychological well-being.

## 1. Introduction

Diabetes mellitus is the most common metabolic disorder in pregnancy, including both pregestational (type 1 or type 2) and gestational diabetes (GDM). Globally, diabetes complicates approximately 1 in 6 pregnancies, with GDM accounting for nearly 86% of cases [1]. Its prevalence has increased substantially over the past two decades with wide variations across regions and populations, reflecting changes in maternal age, obesity rates and screening practices [2]. The substantial economic burden caused by GDM was related to costs associated with increased hospital stay during pregnancy and childbirth [3]. The total costs include direct costs related to medical expenditure (diagnosis, treatment, delivery), direct non-medical costs (traveling, special diet), indirect costs related to loss of productivity because of illness, and intangible costs related to psychological stress [4].

GDM is characterized by glucose intolerance with onset during pregnancy, most commonly in the second or third trimester, and usually resolving after delivery. Established risk factors for GDM include advanced maternal age (over 40), previous GDM, prior macrosomia, family history of type 2 diabetes (T2D), obesity, bariatric surgery, polycystic ovary syndrome, certain ethnic backgrounds (Black, south Asian, Middle Eastern or African-Caribbean origin) [5]. Newer studies have also identified hypertension and environmental and socioeconomic factors as contributors [6,7].

**Figure 1:**
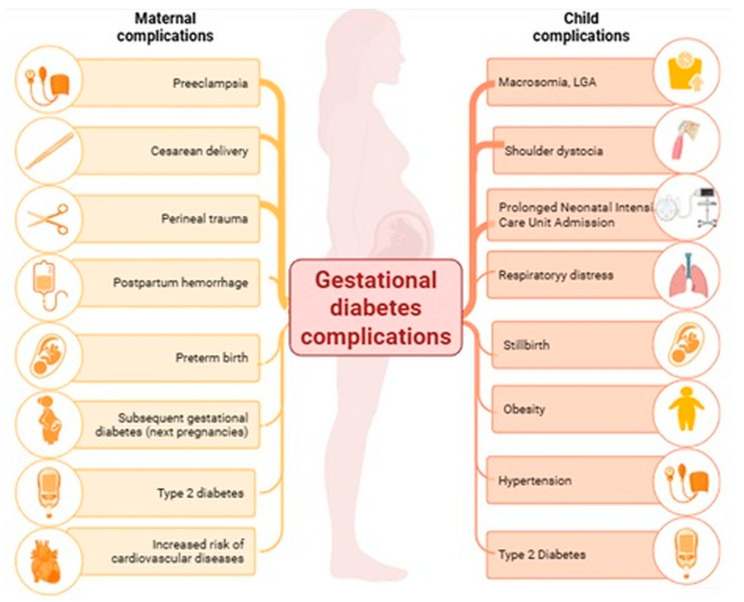
Short- and long-term adverse outcomes of gestational diabetes for mother and child. LGA = large for gestational age.

Although often characterized by mild hyperglycemia, GDM requires close monitoring due to its short- and long-term adverse outcomes for both mother and child (Figure 1). In the perinatal period, it increases the risk of maternal complications such as preeclampsia, cesarean delivery, perineal trauma and postpartum hemorrhage. Neonatal complications include macrosomia, large for gestational age (LGA), shoulder dystocia, respiratory distress, stillbirth and prolonged neonatal intensive care unit admission [8,9,10]. Long-term, women with prior GDM have a 35–50% risk of recurrence and a nearly sevenfold higher risk of developing T2D, while their children face an elevated risk of obesity, hypertension and T2D later in life [11]. Additionally, women with prior GDM have a higher risk of cardiovascular diseases later in life due to significant increase in conventional cardiovascular risk factors, including hypertension, dyslipidemia, and metabolic syndrome [12]. Effective management relies on achieving optimal glycemic control, which is often complicated by the metabolic changes during pregnancy. Early gestation is characterized by a transient increase in insulin sensitivity, followed by progressive insulin resistance during the second and third trimesters, largely driven by placental hormones such as human placental lactogen, progesterone, prolactin, placental growth hormone and cortisol [13,14,15].

Self-monitoring of blood glucose (SMBG) has long been the cornerstone of diabetes management. However, it only provides intermittent measurements and may fail to detect glycemic fluctuations, particularly nocturnal hyperglycemia [16,17,18]. Similarly, glycated hemoglobin A1c (HbA1c), a standard marker for long-term glycemic control, is less reliable during pregnancy due to physiological changes in red blood cell turnover, which result in lower HbA1c levels [19]. Furthermore, HbA1c reflects an average glucose level over time and may not capture postprandial hyperglycemia, a key contributor to complications such as macrosomia [20].

In this context, continuous glucose monitoring (CGM) has emerged as a promising tool in diabetes care. Unlike SMBG, CGM offers continuous, real-time data on glucose trends by measuring interstitial glucose levels at frequent intervals (typically every 1 to 15 min) and transmitting readings to a receiver, insulin pump or smartphone, enabling the evaluation of glycemic variability, time in target range (TIR) and episodes of asymptomatic hypo- and hyperglycemia that might otherwise go undetected. Glucose monitoring techniques include devices broadly categorized into invasive, minimally invasive and non-invasive [21]. Nowadays, minimally invasive devices are the most widely used and there are three types of glucose monitoring systems available: blinded, intermittent and continuous; all of them rely on invasive monitoring techniques by subcutaneous insertion. The most widely used CGM systems are marketed by Medtronic (Galway, Ireland), Dexcom (San Diego, CA, USA) and Abbot (Abbott Park, IL, USA), and differ in terms of lifespan, real-world accuracy, calibration requirements, size, forecasting of glucose trends, alarms for low and high glycemia, improvements for ease of use, option for insulin pump integration, potential interfering substances and price [22,23]. Although all CGM systems rely on the same electrochemical sensing principle, they differ in the way data regarding glucose is delivered to the external devices and read by the patient. Intermittently scanned CGM systems provide data regarding glucose levels only when the user scans the sensor, without automatic alarms. In contrast, real-time CGM systems have incorporated thresholds, rate of glucose changes and predictable alarms for hypo- and hyperglycemia that automatically alert the user even without any user action. For these reasons, real-time CGM is the preferred technology [22,24].

CGM can capture postprandial and nocturnal hyperglycemia, allowing for more precise pharmacologic adjustments and earlier interventions [25,26]. CGM also empowers patients by providing real-time feedback, which helps them understand the impact of food, physical activity and pharmacological therapy on their glucose levels. This feedback promotes lifestyle adjustments, enhances adherence to therapy and reduces the emotional burden of frequent fingerstick testing, ultimately improving the quality of life for women with GDM [27,28,29,30].

Despite the promising potential of CGM and the compelling evidence from studies in pregnant women with type 1 diabetes (T1D) showing improved fetal outcomes with CGM use [31], its role in the management of GDM remains a subject of debate. While some studies report improved metabolic outcomes and a reduced incidence of macrosomia and small for gestational age with CGM use [32], others show no significant advantage over SMBG [33,34,35]. The current evidence is limited, however, by small sample sizes and short time of use of CGM during follow-up. In addition, there are several limitations and challenges regarding the use of CGM in GDM including cost-related accessibility and lack of reimbursement in several countries; limited availability of support for interpretation of large amount of data; discomfort associated with CGM usage (skin reactions, excessive alarms, device operational issues, adhesive problems); and biases among healthcare providers related to knowledge about CGM technology, insurance, and race/ethnicity [36].

## 2. Methodology

This narrative review aims to explore the role of CGM for early detection of dysglycemia, its diagnostic and prognostic value, and its ability to identify specific glycemic patterns during pregnancy complicated with GDM. Furthermore, we aim to assess its role in optimizing lifestyle interventions, as well as its integration into pharmacotherapeutic strategies, by supporting timely initiation, adjustment and effectiveness monitoring of pharmacological therapy. Also, data regarding the integration of CGM with digital health platforms, telemedicine, and Artificial Intelligence (AI)-assisted prediction models in patients with GDM is described here. Finally, we aim to address the psychological impact of CGM, including its potential to reduce the self-perceived stress and enhance the well-being of women with GDM.

Given the emerging topic with heterogenous evidence and the broad scope of this review, we have chosen a narrative approach. This allowed for a wider conceptual framework and greater flexibility compared to strict protocols on a narrow, predefined question specific to systematic reviews. It provided the opportunity to synthetize new ideas on multiple domains (clinical management, patients experience, health economics) and critically review methodologies of available studies on GCM in GDM without requiring exhaustive, reproducible database searches or dual-independent screening.

To address the aims described above, a comprehensive literature search was conducted using PubMed and Embase to identify relevant observational or interventional studies on the use of CGM in GDM. A total of 331 records consisting of original articles, conference papers and other publications were initially retrieved. After removing duplicates and excluding studies that were not directly relevant or lacked full-text access, 74 publications identified during the search were included in this narrative review. Additional relevant studies were identified from previously published meta-analyses, systematic reviews and consensus statements.

## 3. Key Findings from CGM Studies in GDM

### 3.1. Diagnostic and Prognostic Value of CGM in Pregnancies Complicated by GDM

#### 3.1.1. Diagnostic Value of CGM in Pregnancies Complicated by GDM

Several investigators have attempted to identify women at risk for GDM early in pregnancy, but these attempts have faced considerable challenges due to the continuous change in glucose metabolism throughout gestation, a process closely tied to the increasing metabolic demands of the growing fetus [37,38]. Alterations in maternal glucose levels reflect the progressive increase in insulin resistance, specific for the second and third trimesters. Although a dynamic and complex relationship exists between early and later hyperglycemia in pregnancy, conventional diagnostic tools, such as fasting blood glucose and glucose load tests, have not provided sufficient evidence to support cut-off points for GDM diagnosis before 20 weeks of gestation [2]. Carlson et al. evaluated a total of 119 pregnant women undergoing oral glucose tolerance test (OGTT) while blindly being monitored with CGM and found that glucose levels measured with CGM tended to be slightly higher than those measured during OGTT. Thus, this leads to a higher number of women being diagnosed with GDM if OGTT-based thresholds were used during CGM [39]. Nevertheless, the use of fasting glycemia to identify previously undiagnosed T2D in pregnant women with risk factors remains a well-established clinical recommendation [19].

Diagnosis of GDM typically relies on OGTT [19], although evidence points to the role of CGM as complementary to or even a replacement of OGTT. A prospective study evaluated whether glycemic patterns derived from blinded CGM data could characterize pregnancies with GDM (*n* = 768), as diagnosed by standard methods (i.e., OGTT at 24–28 weeks’ gestation). The authors identified distinct CGM-derived glycemic patterns early during pregnancy that were associated with subsequent positive GDM diagnosis at 24–34 weeks. These patterns included higher mean glucose and standard deviation as early as 13 weeks of gestation, a higher coefficient of variation, lower % time in range (TIR) (63–140 mg/dL and 63–120 mg/dL) and lower % time below range (TBR) (<63 mg/dL) with consistently higher mean glucose levels during both daytime and nocturnal periods throughout the gestational period preceding the OGTT. Additionally, the study’s findings suggest that targeting a higher %TIR (63–140 mg/dL) may confer clinical benefit in women with GDM, while a %TIR of 63–120 mg/dL may be more appropriate for pregnant women with normal glucose metabolism. Based on data collected, the authors found that CGM metrics obtained prior to OGTT had the ability to predict GDM, with an area under the receiver operating characteristic curve (AUROC) of 0.81 when using second trimester % time above range (TAR) of 140 mg/dL to predict GDM. The AUROC slightly decreased to 0.74 when %TAR of 140 mg/dL as early as 13–14 weeks of gestation was used to predict GDM [40].

Similar results in terms of predictive performance were reported in two other prospective cohorts. Chen et al. [41] assessed data of 167 pregnant participants who had more than 3 days of CGM data at 18–24 weeks’ gestation and who underwent OGTT at 24–28 weeks. The strongest CGM predictor of GDM was also the %TAR of 140 mg/dL with a cut-point of 1.23%, achieving an AUROC of 0.862 (95% CI: 0.780–0.945), a sensitivity of 80.0%, and a specificity of 84.7%. In a prospective observational study of CGM data collected from 760 pregnant women throughout gestation, the AUROCs for using second trimester %TAR of 140 mg/dL and week 13–14% TAR of 140 mg/dL in predicting GDM were 0.81 and 0.74, respectively [42].

Another prospective study raised the possibility of GDM misdiagnosis when relying solely on OGTT, compared to CGM assessment [43]. In the initial cohort, CGM values at 24–28 weeks of gestation correlated with both 1 h and 2 h OGTT results and identified glycemic variability that OGTT could not detect [44]. A study conducted at Cambridge University Hospitals (UK) further demonstrated that CGM used with or without OGTT was both feasible and acceptable to pregnant women as a diagnostic approach for GDM [45].

#### 3.1.2. Monitoring and Prognostic Value of CGM in Pregnancies Complicated by GDM

Adding 10–14 weeks OGTT or CGM diagnostic criteria for GDM to clinical characteristics improved GDM prediction in a U.S. representative population of over 2000 participants [46]. The GLAM (Glucose Levels Across Maternity) study described the characteristics of CGM profiles in uncomplicated pregnancies (*n* = 413), reporting a TIR (63–120 mg/dL) of 86%, a mean glucose of 98 ± 7 mg/dL, and rare values above 140 mg/dL (2.5%). The authors proposed these values as treatment targets for pregnant women with GDM [47]. Even higher %TIR ranging from 93% to 98% were previously reported in uncomplicated pregnancies [48,49]. GDM is considered to be a temporary illness with remission after birth; CGM data showed that although the glycemic values in GDM cases were comparable with normoglycemic cases during birth, the GDM group had higher mean glucose value and standard deviation, and also higher %TAR (over 140 mg/dL) in the early postpartum period, suggesting that glucose tolerance was not regained immediately after birth [50]. It is estimated that up to one third of GDM cases will have persistent dysglycemia and less than half will perform a postpartum OGTT. Cabrera et al. [51] performed a CGM study in patients with previous GDM, over two periods: immediately after birth and six weeks later. A %TIR (70–180 mg/dL) below 96% predicted abnormal OGTT results with a negative predictive value of 100% and positive predictive value of 54%. Notably, 94% of the participants would prefer as investigation method a CGM over OGTT. In the TOBOGM randomized controlled trial, 1 in 4 women with early GDM (under 20 weeks) had postpartum dysglycemia. The higher weight gain and higher 1 h and 2 h glucose values in the OGTT were independently associated with postpartum dysglycemia [52]. In a retrospective study of 75 GDM women using CGM after diagnosis of GDM, 10.67% developed impaired fasting glucose (IFG) and 9.33% developed impaired glucose tolerance based on postpartum OGTT results. Low blood glucose index, M-value and %TBR were independent predictors of IFG [53].

Although evidence supporting the use of CGM-measured hyperglycemic metrics for GDM prediction and diagnosis is accumulating, it remains limited due to lack of validated diagnostic thresholds compared to OGTT, the variability across devices used for CGM and CGM’s inability to capture glycemic postprandial excursions in a standardized manner [54]. Current clinical guidelines continue to rely on the standard OGTT despite its recognized disadvantages associated with this procedure, including the risk of nausea and vomiting [19]. Given the detailed data it provides, CGM offers increased capability to detect abnormal glucose excursions as early as 13–14 weeks of gestation with good predictive value for GDM that may be missed by conventional testing, thereby improving the potential for better pregnancy outcomes.

### 3.2. CGM-Based Glycemic Targets in Pregnancies Complicated by GDM

Until 2026, in the absence of pregnancy-specific CGM targets for women with GDM or insulin-treated T2D, the American Diabetes Association has endorsed the glycemic metrics proposed by an international consensus panel for pregnant women with T1D [55,56,57]. These standardized parameters provide reference values that may be applied to other forms of diabetes in pregnancy. The threshold of hypoglycemia has not been validated for GDM, but current recommendation is a blood glucose value of below 70 mg/dL and a sensor glucose value of below 63 mg/dL [8]. According to these recommendations, the optimal sensor glucose range during pregnancy is 63–140 mg/dL, with a target of maintaining values within this range for more than 70% of the time (TIR > 70%). Hypoglycemia should be minimized, with fewer than 4% of values below 63 mg/dL (level 1 hypoglycemia) and fewer than 1% below 54 mg/dL (level 2 hypoglycemia). Hyperglycemia or TAR should not exceed 25% of values. For women at low hypoglycemia risk, such as those with GDM managed through diet or T2D with stable glycemic control, a more ambitious target of TIR ≥ 90% is considered both feasible and clinically beneficial, with demonstrated improvements in perinatal outcomes [55,56,57].

More recently, an international consensus statement endorsed by medical societies included recommendations specific for GDM. Although SMBG is recognized as the standard of care, and the evidence for standardized parameters to be provided as reference values in pregnancy and improved outcomes with CGM use in GDM are scarce, this statement recommends for CGM to be offered to pregnant women based on individual preferences and available resources. According to this statement, the optimal sensor glucose range during pregnancy is 63–140 mg/dL with a target of maintaining values within this range for more than 90% of the time (TIR > 90%) and TAR less than 10% [58]. However, observational CGM data collected from normoglycemic pregnant women with normal birth outcomes showed that in physiologic pregnancies the fasting glucose values and postprandial excursions are much lower than the current recommendations for GDM (fasting plasma values of 70–80 mg/dL and 1 h postprandial values 110–130 mg/dL) [59].

Glycemic variability (GV) refers to the fluctuations in interstitial or plasmatic glucose values that occur within and between days that have emerged as a potentially discriminator of risk for adverse maternal and neonatal outcomes, complementing traditional metrics such as TIR. GV metrics derived from CGM is very vast, with over a dozen of parameters being proposed, including: standard deviation (SD) and coefficient of variation (CV) as measure of overall variability relative to mean glucose; mean amplitude of glycemic excursions (MAGE) as a marker of large swings in glucose values; continuous overlapping net glycemic action (CONGA) calculated as the standard deviation of the glycemic changes recorded between a specific point on the CGM profile and a point *n* hours earlier (for example, CONGA 1 h describes short-time, especially postprandial glycemic excursions) [60]; the percentage of mean absolute difference (MAD%) describes how much blood glucose levels fluctuate over a time frame (for example, 72 h), independent of the average glucose level; and inter-day variability measured as the mean of daily differences (MODD) [61]. There are not yet established specific thresholds for these parameters in GDM. However, until specific data is available for GDM, a GV similar to the general population is recommended (for example, a CV below 36%) [31].

GV parameters such as SD and MAGE measured in the first and second trimesters of pregnancy were suggested as potential predictors of subsequent GDM diagnosis, while CV showed a non-significant trend [62]. GV was observed to be higher in GDM than in normoglycemic pregnancies, and diurnal glucose patterns differ throughout the day by 20% in pregnancy versus the nonpregnant state [63]. However, the correlation between GV metrics and other biomarkers for glycemic control failed to be proven in GDM. For example, MAD%, MAGE, SD, CONGA 1–2 values showed no correlation with HbA1c, fructosamine, and 1.5-anhydroglucitol in pregnant women with GDM, showing that those biomarkers could not detect glycemic instability [60,64]. For pregnancy outcomes, there are several studies reporting a correlation between GV parameters and neonatal birth weight and preeclampsia risk, with MAGE, SD, CV, TBR and TAR significantly higher in LGA cases [65,66].

### 3.3. Role of CGM in Lifestyle Optimization in Pregnancies Complicated by GDM

CGM systems enable real-time identification of how lifestyle factors influence glucose values, including food choices, types and duration of physical activity, quality of sleep, and stress. More than ten years ago, it was observed that using CGM data (blinded recordings for three days at that time) to tailor dietary and therapeutic recommendations improved glycemic control. This approach was also associated with a reduced risk of pre-eclampsia, lower rates of primary cesarean delivery, and decreased infant birth weight compared with the standard care [67].

A prospective study evaluated the impact of short-term real-time CGM (3–7 days) within the first 2 weeks after the diagnosis of GDM as an educational tool. While improvements in glycemic variability were observed during CGM use, no significant differences in HbA1c, mean fasting or postprandial glucose levels were noted at the end of pregnancy compared with the SMBG group [34].

The dietary interventions in GDM are challenging. Current recommendations suggest a minimum carbohydrate intake of 175 g/day during pregnancy and maintaining postprandial glucose levels below 140 mg/dL. To reach this goal, daily carbohydrate intake should be divided into three small to moderate sized meals and 2–4 snacks [68]. The use of masked CGM might allow the assessment of usual diets in GDM patients, the effect of dietary pattern and lifestyle components in GDM and the association with different CGM metrics. For example, a secondary analysis of the DiGest trial found that women with GDM consumed a diet low in calories, carbohydrates, and fiber but high in saturated fat. It was also observed that diet and physical activity were not associated with blinded CGM metrics [69].

In a small interventional case–control study enrolling 22 women with mild GDM (81.8% of the cases had fasting glycemia below 95 mg/dL), 72 h CGM data showed that dietary counseling helped women with GDM to achieve glycemic parameters comparable to those of healthy controls [70].

Beyond the total carbohydrate intake, both glycemic index and glycemic load impact postprandial glycemia. Glycemic index was defined as the area under the curve (AUC) in the first two hours after ingestion of carbohydrate-containing food divided by the AUC of the standard reference (either glucose or white bread) and multiplied by 100 [40,71]. Glycemic load was derived from glycemic index and is calculated by multiplying the glycemic index with the grams of carbohydrates available in the food [71]. Despite receiving standardized dietary counseling, only 55% of the pregnant women with GDM in a study adhered to a low glycemic load diet, with carbohydrate intake ranging between 97 and 267 g/day [72]. This variability highlights the need for individualized dietary adjustments, as glycemic responses differ between individuals [68].

Meal composition and timing also influence glycemic control. For example, a cross-over study with 12 women with GDM treated with diet, showed that serving 50% of the daily carbohydrates in the morning increased glycemic variability parameters (MAGE and CV), but improved mean fasting plasma glucose and insulin sensitivity [73].

Using CGM allows pregnant women to experiment with various foods, food combinations and quantities to observe their individual glycemic responses. Carbohydrates are not the only macronutrients correlated with glycemic parameters. An exploratory analysis of CGM recordings and self-reported food intake of 33 GDM women showed that a higher protein intake was associated with lower mean glucose levels and improved glucose AUC, whereas higher carbohydrate and fat intake had a negative impact on AUC, independent of maternal body mass index, age, ethnicity, parity and weeks of gestation [74]. Meal sequencing has also been tested as a strategy to improve glycemic control in GDM. In a CGM study conducted over 15 days and enrolling 27 pregnant women with GDM treated with diet and oral therapy, participants who ate vegetables first, followed by protein and then carbohydrates, experienced a statistically significant reduction in the postprandial glycemia and glycemic variability. This suggests that prioritizing fiber intake could be an effective and simple strategy to improve glycemic control in GDM [75].

Interindividual variability in glycemic responses after a meal may also be partly explained by differences in the gut microbiome. Popova PV et al. [76] conducted a study enrolling 105 pregnant women (*n* = 77 with GDM) who underwent CGM for 7 days. The inclusion in the analysis of microbiome data increased the explained variance in peak postprandial glucose from 34% to 42% [50]. The use of plant-based polysaccharide had the potential to attenuate hyperglycemia and inflammation in diabetic mice models by modulating gut microbiota and alleviating intestinal mucosal barrier damage, but such evidence in GDM in humans is limited [77].

Various dietary patterns have been tested in GDM for achieving the optimal glycemic targets. The eMOM study compared a plant-protein-rich Healthy Nordic Diet (no restriction in carbohydrates intake, *n* = 20) with a moderately carbohydrate restricted diet (around 40% of the calories from carbohydrates, *n* = 22). Both approaches maintained % TIR, with no differences in glucose variability, but the carbohydrates restricted diet resulted in lower mean glucose levels [78]. Emerging digital health solutions, including mobile applications integrated with CGM and AI (e.g., DiaCompanion I), show promise in predicting postprandial glycemic response and are expected to be a successful strategy in reducing the number of clinic visits and increasing favorable maternal and fetal outcomes [79]. Similarly, use of the eMOM app (connected with a CGM, an activity tracker, and food diaries) for 1 week/month was associated with lower maternal fasting and postprandial glucose levels, reduced energy and carbohydrate intake, and increased vegetable consumption [80].

Disruption in normal eating patterns can have a negative impact on glycemic control during pregnancy. A prospective cohort study of 277 healthy pregnant women who underwent CGM recording found that a night eating pattern (more than 50% of calories consumed between 19:00 and 06:59) was associated with higher mean glucose and glucose management index, although it did not increase GDM risk [81].

Although fasting is not recommended during pregnancy due to increased risk of ketosis, it may occur in cultural and religious contexts, such as Ramadan. Studies using CGM in women with GDM who fasted during Ramadan reported improved overall glycemic parameters, but increased time spent in hypoglycemia (by 38.5 min) and frequent post-Iftar hyperglycemic spikes (in 80% of the cases) [82,83]. Despite higher rates of hypoglycemia during fasting, none of the cases required hospitalization or medical assistance for events related to pregnancy. However, data on long-term maternal and neonatal clinical outcomes related to fasting and glycemic status measured by CGM in large cohorts of GDM cases are still required [84].

CGM is particularly valuable in complex clinical situations, such as GDM following bariatric surgery. Case reports highlight its role in the management of complications such as refractory dumping syndrome after Roux-en-Y bypass surgery performed before pregnancy [85].

In a secondary analysis of the DiGest study (Dietary Intervention in Gestational Diabetes), the authors investigated the association of glycemic parameters as assessed by CGM during late pregnancy with breastfeeding outcomes (*n* = 304). The authors showed that favorable breastfeeding outcomes at 3 months postpartum were associated with at least 90% TIR (63–120 mg/dL) and reduced glycemic variability in late pregnancy [86].

The benefits of physical activity in GDM management are more obvious with CGM data. Increased total daily stepping time was significantly correlated with lower 24 h glucose AUC and improved nocturnal glucose levels [72]. Additionally, structured postprandial 20 min interval walks—such as alternating slow and fast intervals—has been shown to reduce postprandial glucose excursions [87].

CGM has also been proven to be an essential tool to investigate the impact of quality and duration of maternal sleep on glycemic parameters in pregnancies complicated with GDM. The use of blinded CGM in 65 GDM women combined with a validated sleep questionnaire (the Pittsburgh Sleep Quality Index) showed that poor sleep quality was associated with a lower proportion of TIR, a greater glucose AUC above target and higher glucose variability [88]. Also, sleep disordered breathing diagnosed by the apnea–hypopnea index was associated with increased glucose levels during the night and persistent elevations into the morning [89].

In conclusion, CGM provides significant benefits in the management of GDM. It supports early identification of the need for pharmacotherapy and timely interventions, improves glycemic control without increasing the risk of hypoglycemia, reduces emergency interventions, and promotes a proactive approach to care. Given the unique physiology of glucose metabolism during pregnancy, predictive models developed for nonpregnant populations are not directly applicable. Advances in AI, mobile applications and integrated digital platforms offer opportunities to combine CGM information with other biological data to enable personalized care and optimize outcomes in GDM [90].

### 3.4. Role of CGM in the Pharmacological Treatment of GDM

Standard management of GDM involves lifestyle modification and, when glycemic targets are not met through diet and exercise alone, pharmacologic therapy such as insulin or metformin is recommended [91]. However, the efficacy of these interventions depends on the accuracy of glucose monitoring. CGM provides detailed information on glucose trends and variability that are particularly useful for the personalization of pharmacologic therapy in GDM.

CGM systems are useful tools for assessing the need for pharmacotherapy and the response to different pharmacological approaches in GDM. Metrics derived from GDM, particularly those reflecting glycemic variability, recorded using CGM, have been shown to predict the need for pharmacological interventions. In a retrospective study of 83 pregnant women with GDM who underwent 6 days of CGM between 26 and 32 weeks of gestation, a higher continuous overlapping of net glycemic action in a period of n-hours (CONGA) and higher mean glucose value (with cut-offs of 86.70 mg/dL, and 98.81 mg/dL, respectively) were associated with the need for pharmacotherapy, demonstrating 83.3% sensitivity and 67.8% specificity [92]. Furthermore, each 1% increment in TAR was associated with a 24% higher probability of requiring pharmacological therapy [93].

Comparative studies further highlight the clinical impact of CGM-guided management. In study of 73 GDM cases (22–34 weeks of gestation, *n* = 36 with CGM and *n* = 37 with SMBG–5 measurements/day), pharmacological therapy (insulin with or without metformin) was initiated in 31% of those monitored with CGM compared to 8% of those with SMBG [94]. The same trend was observed in another study, where 66.7% of the CGM group received insulin therapy and/or metformin, versus 33.4% in the SMBG group. It is interesting that the open-label protocol regarding the use of technology resulted in a significant drop-out from SMBG group and desire to access CGM [95]. These findings suggest that CGM may facilitate earlier and more accurate identification of patients requiring pharmacologic intervention.

The role of CGM was demonstrated in studies evaluating different treatment targets. In a feasibility study exploring the effects and safety of intensive glycemic control (fasting < 90 mg/dL; 1 h postprandial < 120 mg/dL) versus standard targets (fasting < 95 mg/dL; 1 h postprandial < 140 mg/dL) in women with GDM and overweight or obesity, by the end of the trial, pharmacotherapy was prescribed more frequently in the intensive control group than in the standard target group (83% versus 57% of the cases). CGM data showed improved 1 h preprandial, 1 h and 2 h postprandial glucose levels, as well as reduced glycemic excursions in the intensive treated group, without differences in fasting glucose or time spent in hypoglycemia [96].

Beyond guiding treatment initiation, CGM supports individualized pharmacologic strategies by enabling real-time feedback. Pregnant women using CGM are more engaged in their care and can make timely and informed self-adjustments to insulin dosing under clinical supervision. This approach has been associated with more stable glucose profiles and a reduced need for emergency interventions [34]. It was also observed that the use of CGM in insulin treated patients with GDM is associated with a stable HbA1c from week 28 to week 37 of gestation (+1 mmol/mol/0.09%), while in the SMBG group HbA1c had an ascendent trajectory (+3 mmol/mol/0.30%), with significant difference at week 37 between groups in the favor of the CGM group [97]. The ability to accurately visualize glucose trends allows both patients and healthcare providers to proactively modify pharmacologic therapy to prevent glycemic excursions rather than reactively addressing hyperglycemia. Importantly, CGM can be integrated with other digital health apps such as closed-loop control systems, insulin titration applications, nutrition tracking tools and physical activity monitors. Together, these systems offer opportunities to improve the quality of care for women with diabetes in pregnancy [98]. Overall, CGM may represent a paradigm shift in the pharmacological management of GDM transitioning from reactive, intermittent adjustments to proactive, data-driven, individualized interventions.

### 3.5. Role of CGM in Improving Glycemic Control, Maternal and Neonatal Outcomes in GDM

Convincing evidence supports the use of CGM during pregnancy in women with T1D, demonstrating improved glycemic control, as well as maternal and neonatal outcomes [99,100]. These data have led international guidelines to recommend CGM in T1D both preconception and throughout pregnancy in this population [19,101,102]. In addition to clinical metrics, CGM use has been associated with enhanced patient confidence and reduced anxiety, particularly when glucose data can be shared with healthcare providers or accessed via smartphone applications [18]. These psychosocial benefits may further improve adherence to pharmacological therapy and contribute to favorable perinatal outcomes.

In GDM, evidence on the improved outcomes associated with CGM use remains limited and inconsistent. Available studies report conflicting results, and the efficacy of CGM in improving glycemic control and pregnancy outcomes in this population is still being assessed. Several methodological limitations may explain the lack of consistency observed across these studies. Relatively small sample sizes (often fewer than 200 participants) as well as short CGM recordings (limited to several days or weeks after GDM diagnosis) may have limited a comprehensive assessment and increased the risk of a type II error. Also, inconsistent findings may result from differences in GDM definitions used for participant selection (International Association of the Diabetes and Pregnancy Study Groups, local guidelines or criteria for high-risk groups or American Diabetes Association) and gestational age at enrollment, both of which could have influenced the observed association with maternal and neonatal outcomes. Importantly, heterogeneity among studies may also reflect differences in CGM device types (real-time vs. intermittently scanned) and glucose targets used for therapeutic management [32,33,34,35,67,83,97,99,103,104,105].

In this section, we review the available evidence and discuss its implications for the use of CGM after GDM diagnosis. Additional details from these studies are provided in Supplementary Table S1.

#### 3.5.1. CGM Use, Glycemic Control, and Risk of Hypoglycemia in GDM

The use of CGM in GDM has been associated with improved detection of hyper- and hypoglycemia, and better glycemic control across both observational and randomized controlled studies.

Prospective observational studies showed that CGM can identify unrecognized postprandial hyperglycemia, nocturnal hypoglycemia and glycemic excursions that may be missed by intermittent SMBG, resulting in improved glycemic control and prompting therapy change [25,83,103,104]. The largest observational prospective study reporting glycemic control in GDM was performed in 350 pregnant women. It showed that retrospective CGM use for 4 weeks after the diagnosis (at 24–28 weeks of gestation) reduced glycemic variability and shortened the duration of hyperglycemia or hypoglycemia compared with baseline (*p* < 0.001). Compared with SMBG, CGM improved variability metrics, including standard deviation of sensor glucose, mean amplitude of glycemic excursions (MAGE), and mean of daily differences (*p* < 0.001) while also reducing hypoglycemia duration. Notably, only 3.4% of women using CGM experienced hypoglycemia with a duration of more than 30 min/day, compared to 19.4% of the women on SMBG routine care (*p* < 0.001) [67]. Additionally, among pregnant women with insulin-requiring GDM, CGM use was associated with a significantly shorter time to reach glycemic targets, fewer hypoglycemic events, and improved glycemic variability [67].

Evidence from randomized clinical trials remains mixed in GDM. While several studies have not reported HbA1c improvements with CGM use, improved glycemic variability and reduced hypoglycemia frequency were more consistently reported. In a randomized study of 106 pregnant women with GDM, CGM use (early vs. late in pregnancy) showed a non-significant decrease in HbA1c in the CGM group (5.5% vs. 5.6%) with no difference between early and late initiation [33]. Early CGM compared to late use improved glycemic variability, as measured by MAGE, although no difference was observed in mean sensor glucose and other CGM-derived metrics [33]. Similarly, three other studies reported comparable HbA1c values with real-time and retrospective CGM and SMBG [34,94,105]. In one of these studies, a significant improvement in the parameters of glucose variability was observed on the last day of sensor application; both mean glucose and the standard deviation of mean glycemia decreased despite no difference in the HbA1c [34]. Another trial demonstrated improved HbA1c by 37 weeks of gestation with intermittent retrospective CGM (6-day sensor) at 28, 32 and 36 weeks of gestation compared to usual care including SMBG [97]. Despite variability in individual trials results, two meta-analyses of four and five randomized controlled trials, respectively, reported a modest but statistically significant reduction in HbA1c with CGM compared to SMBG (mean difference ranging between −0.18% and −0.21%) [99,106]. Also, intermittent scanning CGM has been shown to significantly reduce the frequency of hypoglycemia as shown by another randomized controlled trial enrolling 110 women with GDM [107].

#### 3.5.2. CGM Use and Maternal Outcomes in GDM

While reduced TIR and increased glucose variability during pregnancy have been associated with adverse outcomes in women with T1D or T2D [31], such as higher risk of hypertensive disorders of pregnancy, preeclampsia, preterm delivery, and cesarean delivery, their correlation with pregnancy outcomes in GDM remains uncertain. Evidence is limited, and available studies report heterogeneous findings.

Observational studies that did not investigate CGM-derived metrics have not demonstrated a clear benefit of CGM use on maternal outcomes. A recent retrospective analysis of 277 women with GDM (224 using SMBG and 53 flash glucose monitoring) reported higher weight gain in the CGM group than in the SMBG group (12 kg vs. 10 kg) without differences between groups in obstetric outcomes such as cesarean section, preterm birth, gestational age at delivery, birth weight or perinatal complications [108]. Similarly, a pilot prospective study comparing flash CGM with SMBG from diagnosis until delivery found no significant difference in the maternal outcomes between study groups [109].

In contrast, studies evaluating CGM metrics such as %TIR or glycemic variability as assessed by MAGE suggest potential association with improved outcomes. Retrospective data from 65 pregnant women with T2D or GDM showed that a TIR ≤ 70% was associated with a higher risk of hypertensive disorders of pregnancy (odds ratio [OR] 3.9; 95% confidence interval [CI] 1.3–13.0), preterm delivery (OR 3.1; 95% CI 1.1–9.1), and cesarean delivery (OR 4.6; 95% CI 2.2–15.1) compared with a TIR > 70% [110]. Similarly, a large prospective observational study of 760 women with GDM showed that 2nd trimester %TIR had a good predictive accuracy and specificity for hypertensive disorders of pregnancy [42].

These later findings are supported by the experimental prospective data. The study of Yu et al. [67] performed in 350 pregnant women with GDM showed that retrospective CGM use for 4 weeks after GDM diagnosis (at 24–28 weeks of gestation) reduced the incidence of preeclampsia (3.4% vs. 10.1%) and cesarean delivery (34.7% vs. 46.6%). Furthermore, glycemic variability, as assessed by MAGE, improved with retrospective CGM use and was independently associated with preeclampsia risk (OR 3.66; 95% CI 2.16–6.20, *p* < 0.001), but not with cesarean delivery (OR 1.27; 95% CI 0.96–1.68, *p* = 0.101) [67]. Additionally, the GlucoMOMS open-label randomized study, which enrolled 300 women with T1D, T2D or GDM treated with insulin, reported a reduced risk of preeclampsia in the GDM subgroup using retrospective CGM compared with SMBG (relative risk [RR] 0.14; 95% CI 0.02–1.12, *p* = 0.03) [111]. However, other analyses have not identified associations between CGM metrics (TIR, TAR, TBR, mean standard deviation of the sensor average glucose) and preeclampsia, irrespective of the pregnancy trimester analyzed [112].

In contrast with these results, small randomized controlled studies employing intermittent CGM use after GDM diagnosis at 24–28 weeks of gestation failed to identify any association of CGM use or CGM-derived metrics with maternal obstetric outcomes [32,33,34,35,94,97]. Nevertheless, two of these studies reported a lower proportion of excessive gestational weight gain among CGM users [33,34]. These findings are consistent with a recent meta-analysis of seven randomized controlled trials (597 participants), which showed no significant reduction in cesarean delivery rates with CGM use (OR 0.93; 95% CI 0.67–1.29) [99].

In summary, current evidence, most of which is of low certainty, suggests that CGM use alone may have a limited impact on maternal outcomes in GDM. However, improved CGM metrics, such higher %TIR, lower %TAR, and lower MAGE may be associated with better maternal outcomes, including lower risk of hypertensive disorders and preeclampsia. Larger randomized controlled trials evaluating CGM metrics longitudinally from the time of GDM diagnosis are needed to clarify these associations.

#### 3.5.3. CGM Use and Neonatal Outcomes in GDM

Although compelling evidence that proved the beneficial effect on HbA1c of CGM use throughout the pregnancy translating to improved neonatal outcomes are available for T1D [19,99,101,102], the benefits are less clearly established in GDM with both positive and neutral effects being reported from clinical studies.

Several small sample observational studies explored the relationship of CGM use and CGM-derived metrics with neonatal outcomes and demonstrated that higher mean sensor glucose and overnight sensor glucose were associated with birth weight and LGA occurrence. An observational study of 97 women who underwent CGM for 5–14 days at a mean of 28.8 gestational weeks showed that each 1-SD increase in maternal nighttime mean sensor glucose and severe variability glucose mode were associated with 6.0 (95% CI 0.4–11.5) and 6.3 (95% CI 0.4–12.2) percentage points increase in birth weight percentile [113]. Similarly, a study of 162 women with GDM using 7-day masked CGM at 30–32 weeks’ gestation revealed that mothers of LGA infants had significantly higher mean sensor glucose driven by nocturnal glucose as compared to those with no LGA, while there was no significant difference in TIR, TAR, and TBR between groups [114].

More recently, larger observational studies confirmed the relationship of CGM-derived metrics with neonatal outcomes. The same study by Yu et al. [67] described above, assessing data from 336 Chinese women and 332 newborns, showed that intermittent masked CGM use for 4 weeks was associated with a lower frequency of premature delivery, a lower frequency of macrosomia, of LGA, hyperbilirubinemia, hypoglycemia in the newborn and the need for supplemental oxygen in the neonatal nursery beyond 4 h after birth as compared with SMBG (*p* < 0.05 for all neonatal outcomes). MAGE was an independent predictor for macrosomia (OR, 1.90; 95% CI 1.19–3.04), neonatal hypoglycemia (OR, 1.63; 95% CI 1.07–2.48), neonatal nursery beyond 4 h after birth (OR 1.73; 95% CI 1.04–2.90) and a composite neonatal outcome (OR, 1.34; 95% CI 1.01–1.77). Additionally, sensor glucose was an independent predictor of LGA and small for gestational age, neonatal nursery beyond 4 h after birth, and a composite neonatal outcome [67]. Similarly, in the largest prospective observational study, enrolling 1302 Chinese women with GDM who underwent 14-day masked GCM after the recruitment at 24–28 weeks of gestation, TAR, glucose AUC, nighttime mean sensor glucose, daytime mean sensor glucose, and daily mean sensor glucose were associated with a higher risk of any adverse neonatal outcome, with OR: 1.22 (95% CI 1.08–1.36), 1.22 (95% CI 1.09–1.37), 1.18 (95% CI 1.05–1.32), 1.21 (95% CI 1.07–1.35), and 1.22 (95% CI 1.09–1.37), respectively. In addition, TIR, TAR, AUC nighttime mean sensor glucose, daytime mean sensor glucose, and MAGE were positively associated with a higher risk for LGA, while TBR was negatively associated with the risk of LGA. Also, a higher TAR was associated with a higher risk of neonatal intensive care unit admission (OR 1.24; 95% CI 1.07–1.44]) [115].

Among the randomized controlled studies assessing CGM use in GDM, only a few reported the association of CGM metrics with neonatal outcomes. The FLAMINGO trial was a randomized controlled trial in which 100 pregnant women with GDM were recruited between 24 and 28 weeks of gestation and were randomly assigned to either flash glucose monitoring for 4 weeks or SMBG. The results showed a significantly higher incidence of fetal macrosomia in the SMBG group than in the CGMS one (20% vs. 4.08%; OR 5.62; 95% CI 1.16–27.22). There was no statistically significant difference between study groups for LGA and neonatal hypoglycemia. Neither mean fasting nor postprandial sensor glucose was associated with fetal birth weight, but postprandial sensor glucose was correlated with the incidence of fetal macrosomia (ROC AUC 0.704; 95% CI 0.546–0.862) [32]. Similar results were recently reported in the Steady Sugar trial. This was also a randomized controlled trial in which 120 women with GDM were recruited before 26 weeks of gestation and randomized to either continuous CGM or SMBG plus monthly blinded CGM. The results showed a significantly higher gestational age at delivery, lower preterm delivery rates, lower incidence of LGA and fewer neonatal intensive care unit admissions with CGM. Mean glucose and TAR were negatively correlated with gestational age at birth (ρ = −0.25, *p* = 0.027 for mean glucose; ρ = −0.23, *p* = 0.043 for TAR) [116]. Seven other randomized controlled studies assessing the effect of intermittent CGM use vs. SMBG in GDM reported no difference between groups in neonatal outcomes (preterm births, macrosomia, hyperbilirubinemia, neonatal hypoglycemia, respiratory distress, and neonatal intensive care unit admission >24 h) [33,34,35,94,97,111]. However, a lower newborn birth weight was observed in the CGM group (3123.79 ± 369.58 g in the CGM group vs. 3291.56 ± 386.59 g in the SMBG group, *p* = 0.015) in one of these studies [112]. These observations were confirmed by the GRACE trial which showed that CGM use in GDM reduces LGA rates, but the tight glycemic control was associated with a higher-than-expected overall prevalence of small for gestational age infants, requiring further research [117].

However, the results of meta-analyses are not consistent. A meta-analysis of six randomized controlled studies assessing the outcome of 510 pregnancies showed that CGM use vs. SMBG was not associated with reduced LGA (OR 0.73; 95% CI 0.45–1.19), neonatal hypoglycemia (OR 0.77; 95% CI 0.44–1.36) or neonatal intensive care unit rates (OR 1.12; 95% CI 0.63–1.99) [99]. A recent meta-analysis of five randomized controlled studies including 475 participants showed a lower risk of macrosomia (OR 0.5; 95% CI 0.040–0.61) and lower birth weight (mean difference −151.68 g) with CGM use when compared to SMBG. No difference in the risk of neonatal hypoglycemia, preterm delivery or admission to the neonatal intensive care unit were found [106]. The largest meta-analysis to date including 17 randomized controlled studies and 2349 women with GDM showed that CGM use was associated with significantly lower risk of neonatal intensive care unit admission compared with SMBG (RR 0.75; 95% CI 0.59–0.97) and a non-significant trend toward reduced incidence of LGA (RR 0.81; 95% CI 0.65–1.01) and neonatal hypoglycemia (RR 0.85; 95% CI 0.70–1.03). Macrosomia risk was not assessed [118].

Real-time vs. blinded CGM does not appear to influence the outcomes—in another study including 23 women with GDM, the lack of difference in the CGM metrics between groups (TIR, TAR, TBR, mean sensor glucose) translated into the lack of difference between study groups for neonatal outcomes [105].

In conclusion, we found evidence that CGM use in GDM is probably associated with improved neonatal outcomes, reducing the risk of macrosomia and LGA in the newborn as compared to the SMBG. Mean overall sensor glucose, nighttime sensor glucose, and lower MAGE may be associated with improved neonatal outcomes but, as for maternal outcomes, larger randomized controlled trials are required.

### 3.6. Role of CGM Use in Reducing Blood Glucose Monitoring-Associated Stress in GDM

Limited research on the psychological consequences of GDM diagnosis is available, including the stress and challenges associated with its management, and even fewer studies have examined the potential of technology to alleviate this burden. The quality of life (QoL) in women with GDM covers various aspects of an individual’s well-being, including physical and mental health, social functioning, and disease management [119]. Women diagnosed with GDM often come across many challenges, such as following a strict diet, frequent blood glucose monitoring, and in some cases, starting insulin therapy [120]. These burdens can significantly increase psychological stress, anxiety, and frustration, particularly when it interferes with daily life, reducing the overall QoL among women with GDM, with approximately a quarter reporting a poor QoL [121]. Previous studies have also shown that women with GDM have experienced feelings of failure and powerlessness along with GDM diagnosis [122,123]. However, women with GDM experience a rapid and intensive learning process, quickly passing from the immediate emotional impact of receiving the diagnosis to a phase of proactive disease management [124].

To alleviate the burden and the stress that comes together with GDM, it is important to understand the management, therapeutic goals, and associated risks. CGM devices are significant tools to address glycemic rises and falls, using either lifestyle optimization or pharmacological interventions. By eliminating the repetitive discomfort related to finger-prick testing and replacing it with automated CGM throughout daytime and nighttime, CGM reduces both physical and psychological burden. The benefits that come along with the use of the applications dedicated to each device ease patient–doctor communication and reduce the time dedicated to fill in glycemic journals. Improvements in technology have significantly reduced the need for manual data entry, as CGM enables automatic and more frequent glucose measurements [125]. Moreover, CGMs have been shown to be well-accepted among women with GDM [126].

Miremberg et al. reported improvements in glycemic control in women with GDM by providing personalized medical feedback [127]. In contrast, mHealth interventions with reduced medical input show limited efficacy [128]. Augmenting these tools with real-time sensor data readings may support self-discovery by helping women understand lifestyle–glucose associations [129]. In a study conducted by Kytö et al. [130], self-discovery using CGM facilitated participants’ understanding of the relationship between blood glucose levels and nutritional intake. Regarding user experience, participants reported that the perceived benefits outweighed the discomfort and effort associated with the sensor.

In a study by Won et al., the use of intermittently scanned CGM significantly improved treatment satisfaction (overall score: 10.36 ± 9.21, *p* < 0.001), although perceptions of hypo- and hyperglycemia remained largely unchanged. GDM was shown to significantly reduce QoL in nearly 90% of the 189 women surveyed. All 19 domains assessed using the Korean version of the Audit of Diabetes-Dependent Quality of Life Questionnaire (K-ADDQoL) were negatively impacted. The domain most severely affected was freedom to eat (−6.98 ± 2.49, *p* < 0.001), while sex life was the least affected (−0.25 ± 0.80, *p* = 0.008). The greatest reduction in QoL was observed among younger women and those undergoing insulin therapy. Interestingly, women who perceived less QoL impairment due to GDM had a higher increase in HbA1c levels one year after delivery (difference in HbA1c: 0.3 ± 0.4%) compared to those who had a more important QoL impairment (0.0 ± 0.4%) [131].

Bastobbe et al. [109] enrolled 37 women with GDM using intermittently scanned CGM and compared them to 74 pregnant women using SMBG and reported increased convenience (mean: 2.61), flexibility (mean: 2.56), treatment satisfaction (mean: 1.88), and understanding of GDM (mean: 1.5) in those using CGM. They were also likely to recommend CGM for other pregnancies with GDM. Perceptions of hyperglycemia and hypoglycemia remained unchanged. In a randomized controlled trial conducted by Lane et al., women with GDM using real-time CGM reported that real-time feedback enabled them to take better dietary decisions. Notably, 90% of participants felt that the advantages of this continuous data offered by the real-time CGM were considered more beneficial than the inconvenience of wearing the device [105]. In the DipGluMo Study, 302 pregnant women with GDM were randomly assigned to real-time CGM or SMBG. Although the primary endpoint (a composite of perinatal outcomes: LGA, macrosomia, polyhydramnios, neonatal hypoglycemia, and stillbirth) was not influenced using CGM, after giving birth, the participants were asked to complete a satisfaction questionnaire regarding the glucose monitoring methods. The responses showed that in both groups the participants would prefer the CGM method if they had the choice and participants from CGM group found the use of the sensor significantly easier than SMBG [132].

Previous studies, including populations with T1D, reported significant improvements in hypoglycemic confidence (*p* = 0.01) and reductions in diabetes-related distress (*p* = 0.01), compared to the SMBG group, while satisfaction with CGM use was positively associated with decreased hypoglycemic fear (*p* = 0.02) and decreased diabetes distress (*p* < 0.001) [133]. Nonetheless, a study conducted in Belgium, involving 17 different medical centers, that included 515 patients with T1D that have received real-time CGM, reported that QoL and work absenteeism improved and fear for hypoglycemia decreased [134]. Although these studies did not assess women with GDM, we can assume similar positive effects of CGM on fear of hypoglycemia and work absenteeism.

Real-time visualization of glycemic patterns using CGM enhances GDM patients’ engagement and self-efficacy, helping them make informed dietary and therapy decisions without the uncertainty generated by isolated SMBG readings [135]. On the other hand, alarm fatigue generated by frequent alerts, data overload, and anxiety related to real-time monitoring blood glucose excursions could potentially counteract the previously described benefits of using CGM devices [136].

The limitations encountered in the studies described in this chapter were small cohorts, sub-optimal adherence to device requirements, use of sometimes now-outdated devices and in the short term.

**Figure 2:**
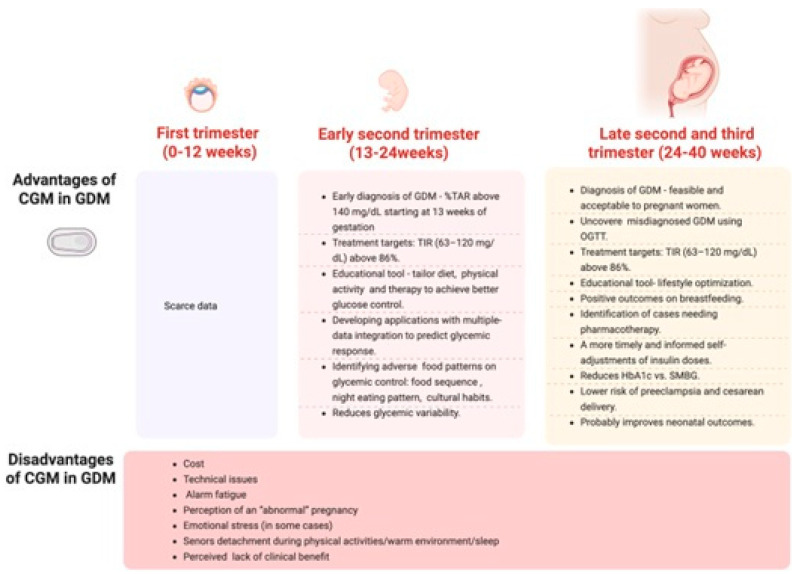
Advantages and disadvantages of continuous glucose monitoring (CGM) in gestational diabetes (GDM). OGTT = oral glucose tolerance test; TIR = time in range; HbA1c = glycated hemoglobin.

The advantages and disadvantages of using CGM in GDM are presented in Figure 2.

### 3.7. Barriers to Initiating CGM During GDM

Despite the recognized clinical benefits of CGM, several barriers limit its widespread usage during GDM. One of the most frequent challenges remains the elevated cost, with studies showing that CGM-associated expenses were nearly three times higher than those of SMBG [112]. Although in a cost–consequences analysis using real-world data in pregnant women with T1DM the cost of CGM was offset by better neonatal health outcomes [137], such evidence is not yet available for GDM [138].

Psychosocial factors also contribute to resistance or discontinuation. For some women, the presence of wearable sensors gave the perception of an abnormal pregnancy, intensifying emotional stress and reducing acceptance of the technology [111]. Still, pregnant participants showed high tolerability in wearing CGM devices for up to 123 days [75].

Technical limitations further complicate the user experience. The use of SMBG is recommended for glucose monitoring in pregnancy. A study investigating the accuracy of glucometers in pregnancy obtained >98–99% of the meter values in the acceptable zones of the error grid for the majority of the devices, but there was a mean difference between glucometer and laboratory value of −0.33 mmol/L (−6 mg/dL) and 0.73 mmol/L (13 mg/dL) [139]. Discrepancies between CGM and capillary glucose measurements created confusion about data reliability. Therefore, is important to train patients before CGM use and explain the significance of the value (glucose from the interstitial fluid and not blood glucose), lag time of a few minutes between blood glucose readings and CGM values, especially during high excursions of glycemia and acceptable difference of 20% at any given time versus capillary glycemia [140].

Sensor placement issues also raise a practical challenge; while most users preferred arm placement over the abdomen for comfort, this location was associated with false low alarms during sleep and required assistance for application, affecting autonomy and long-term adherence. Additionally, sensors frequently detach during physical activities [130].

Additional data on potential barriers in CGM use are derived from studies performed in T1D. In a cohort study including pregnant women with T1D, although 90% of participants continued real-time CGM after six months, discontinuation in some cases was attributed to system-related stress, psychiatric comorbidities, or termination of reimbursement following delivery [134]. Similarly, CGM usage was refused with alarm fatigue, technical issues, and perceived lack of clinical benefit reported as common reasons for discontinuation [141].

**Figure 3:**
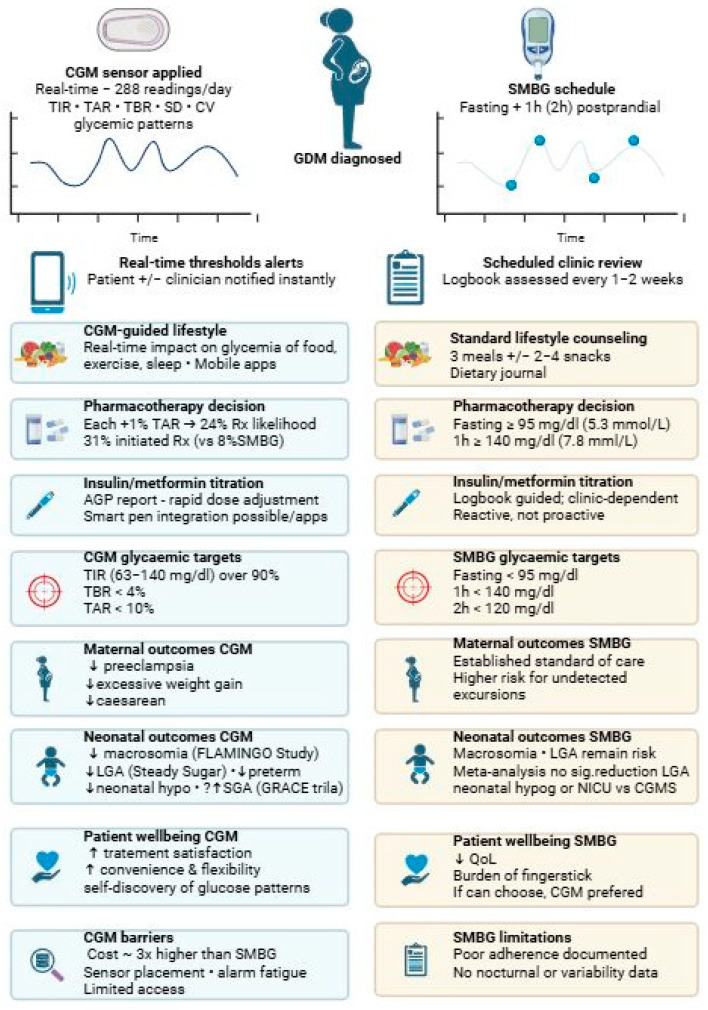
Comparison of continuous glucose monitoring (CGM)-guided management pathway versus self-monitoring blood glucose (SMBG)-guided pathway in gestational diabetes mellitus (GDM). NICU = neonatal intensive care unit; QOL = quality of life; TIR = time in range; TBR = time below range; TAR = time above range; SD = standard deviation; CV = coefficient of variation; LGA = large for gestational age.

Nonetheless, to improve user engagement, there is a growing call for more user-friendly sensor designs, and the integration of personalized feedback mechanisms or AI-driven models to support behavioral adaptation and improve the user experience [142]. A schematic illustration comparing the CGM-guided management pathway with SMBG-guided pathway is presented in Figure 3.

### 3.8. Integration of CGM with Digital Health Platforms, Telemedicine, and AI-Assisted Prediction Models in GDM

Data provided by CGM is embedded within digital health ecosystems such as smartphones (through mobile applications) and cloud-based dashboards (web-accessible platforms that aggregate and monitor healthcare data), enabling real-time patient feedback and healthcare provider access. Smartphone applications designed for women with GDM were proven to significantly lower overall glycemic pre- and postprandial parameters and were non-inferior in adverse maternal and child outcomes, reflecting the benefits of the application in terms of education, motivation and self-management empowerment [143,144]. A randomized clinical trial including patients with GDM (*n* = 106) was conducted by Munda et al. to assess the outcomes of telemedicine care compared to standard care. The telemedicine group evaluated using a mobile application installed on the smartphone and monthly video calls presented with less postprandial glucose levels above the target, lower cesarean section rate and higher patient satisfaction compared to the standard group [145]. The results of a meta-analysis including 12 randomized control trials evaluating telemedicine versus standard care for GDM (*n* = 2192) confirmed the significant difference in postprandial blood glucose control and cesarian section effect rate, although there was no significant effect on HbA1c, preterm birth or fetal macrosomia [146].

Machine learning algorithms are being increasingly used to analyze data monitored using CGM. Kokori et al. performed a review to compile available studies investigating the application of machine learning for first trimester GDM risk prediction. Several readily available clinical characteristics, such as body mass index, fasting blood glucose and maternal age were reported as significant predictors, although the need for tailoring predictive models in specific populations and groups was underscored by authors [147].

Clinical evidence supports the use of telemedicine integration in the management of patients with GDM and the use of AI-assisted risk prediction models for GDM. However, there is still uncertainty persisting over the value of CGM integration in GDM that might be conditioned by CGM validation as a diagnostic, monitoring and prognostic tool in pregnant women with GDM.

## 4. Perspectives for Clinical Practice

Despite increasing interest in CGM, current evidence regarding its role in the management of GDM remains limited by small sample sizes and a low number of studies. Larger clinical trials are needed to fully understand the benefits of CGM in GDM, as well as its impact on pregnancy outcomes, glycemic control and psychological well-being. Future research, particularly studies integrating digital health solutions, is expected to provide further insight into the long-term benefits, usability, and cost-effectiveness of CGM in pregnancies complicated by diabetes [99].

CGM is emerging as a clinically powerful, patient-preferred tool for early dysglycemia detection in pregnancy (as early as 13–14 weeks of gestation), with predictive values comparable to those of OGTT for GDM prediction, and unique capabilities in capturing nocturnal hyperglycemia and glucose variability-related risk that SMBG or other tests cannot match. However, the main remaining gap is prospective outcome data linking early CGM-detected dysglycemia to improved perinatal outcomes. In the absence of these prospective studies, we do not know whether diagnosing and treating GDM in early pregnancy can improve perinatal outcomes [2].

In terms of maternal benefits, the most consistently demonstrated one is improved glycemic control, reflected by the lower HbA1c levels at the end of pregnancy. Meta-analyses of randomized controlled trials showed that women using CGM achieved significantly lower HbA1c compared with those using SMBG alone, with reductions of approximately 0.2% [106]. CGM allowed early identification of hyperglycemic patterns, facilitating more precise titration and timing of pharmacotherapy. Its use between 24 and 32 weeks of gestation has been associated with fewer undetected episodes of hyperglycemia and nocturnal hypoglycemia, allowing a better and more effective therapy adjustment [27]. Moreover, CGM improves the detection and management of nocturnal glucose excursions. Nocturnal hyperglycemia, often asymptomatic and not detected by SMBG, can be easily identified with CGM. This is critical, as nocturnal hyperglycemia has been associated with an increased risk of adverse pregnancy outcomes [27]. Additionally, evidence is available on the reduced maternal glycemic variability with CGM use, with improved TIR, and reduced TAR and daily glucose fluctuations [148]. Data regarding CGM-derived metrics, such as TIR, TAR, TBR, MACE, SD, CONGA, GV, CV, and pattern recognition were described in several studies and were useful in informing and providing support for individualized treatment adjustments in pregnant women with GDM, going beyond conventional glucose monitoring approaches. Consequently, these led to lower maternal glucose exposure, which may subsequently improve both maternal and neonatal outcomes.

For other maternal outcomes, current evidence is limited both in quality and quantity and with several studies reporting lower risk of hypertensive disorders and preeclampsia associated with improved glycemic exposure due to CGM use, thus probably reducing the risk of these specific outcomes. However, despite the theoretical benefits, current evidence does not demonstrate a consistent and significant reduction in cesarean section rates and preterm birth [99,106,148,149]. Larger randomized controlled trials prospectively evaluating the effect of improved glucose metrics with CGM use are needed to clarify these associations.

CGM-guided management of GDM appears to improve several neonatal outcomes, by reducing fetal exposure to maternal hyperglycemia. A lower birth weight and reduction in LGA infants and macrosomia are the most consistently reported benefits of CGM-guided management of GDM. Studies have shown that pregnant women using CGM have a significantly lower likelihood of delivering infants whose birth weight exceeds the 90th percentile for gestational age [106,118,148]. Additional benefits may include a reduction in neonatal hypoglycemia and neonatal intensive care unit admission with CGM use during pregnancy, although improvement of these outcomes is not consistently reported across studies [118,150]. The mechanism explaining these improved outcomes is related to improved maternal glucose control, reduced fetal hyperglycemia and hyperinsulinemia and subsequent reduced fetal overgrowth [54]. These findings are in agreement with prior data highlighting the importance of tight glycemic control, particularly in the postprandial period, in preventing fetal macrosomia and related complications, such as birth trauma and shoulder dystocia [151]. Despite the above-mentioned neonatal benefits, current evidence does not demonstrate significant reductions in preterm birth, respiratory distress syndrome, or perinatal mortality.

Despite the benefits, CGM adoption in GDM care comes with several challenges. High cost of devices, lack of reimbursement, and limited availability in low-income countries remain significant barriers. Access is often limited by reimbursement policies and insurance coverage, creating disparities in utilization between healthcare systems and patient populations. These challenges are particularly relevant for low- and middle-income countries and healthcare systems with limited resources. High device costs, limited reimbursement in terms of amount or populational groups, supply-chain constraints, inadequate digital infrastructure, and limited number of healthcare professionals trained in diabetes technology may all limit CGM adoption. In such resource-limited settings, selective use of CGM in pregnant women requiring insulin therapy or those with persistent hyperglycemia despite standard management may represent a more economically sustainable approach [101].

Technical issues, such as calibration errors or improper sensor placement, may compromise effectiveness. Insufficient training of both healthcare providers and patients, or the patient’s inability to properly manage these devices due to their health literacy, can hinder optimal use. Integration of CGM data into routine clinical practice, along with supportive follow-up, is essential to maximize benefits. Furthermore, the current evidence is disproportionately derived from high-income settings, limiting its generalizability. There is an urgent need for implementation research in low- and middle-income countries, where GDM prevalence is rising, and healthcare access remains variable [18].

Although this narrative review provides a novel up-to-date perspective on the potential benefits of CGM integration into personalized therapeutic decision-making during pregnancy, it has several limitations. Although it synthesizes a broad range of data, it is a non-systematic review and does not include an assessment of study quality. However, the studies included were published in respected journals within the past decade. The review reflects a multidisciplinary perspective and aims to provide a comprehensive overview of the current state of knowledge in this field. Further research, including more rigorous methodologies, such as systematic reviews and meta-analyses, would allow for more robust and precise conclusions.

## 5. Conclusions

In conclusion, CGM offers a promising alternative to traditional methods such as SMBG or HbA1c testing, providing real-time data and enhancing both the efficacy of clinical management and patient empowerment. CGM-derived metrics provide additional data potentially allowing the integration of CGM into personalized GDM treatment decisions, beyond the conventional glucose monitoring approach. Furthermore, integration of CGM data with telemedicine and AI-assisted prediction models in GDM offers additional benefits. However, costs and access remain the main barriers in CGM utilization and current evidence is still limited to support the integration of CGM into the routine care of all pregnant women with GDM, as part of a comprehensive and individualized treatment strategy. Larger, multicenter clinical trials with CGM use from the GDM diagnosis to delivery are needed to fully understand the benefits and optimal use of CGM in GDM, as well as its impact on pregnancy outcomes, glycemic control, and psychological well-being.

## Data Availability

No new data were created or analyzed in this study.

## Supplementary Materials

The following supporting information can be downloaded at https://www.mdpi.com/article/10.3390/diagnostics16142145/s1, Table S1: Selected studies using CGM in gestational diabetes and their main results.
