# Supplementary material for: Managing Gestational Diabetes Complexity with Continuous Glucose Monitoring: A Narrative Review
Source: Diagnostics (Basel). 2026 Jul 8;16(14):2145. doi: 10.3390/diagnostics16142145 (PMC13407910; doi:10.3390/diagnostics16142145)
Supplement: Supplementary file 1 [file diagnostics-16-02145-s001.zip › diagnostics-4331957-supplementary.pdf]

Table S1. Selected studies using CGM in gestational diabetes and their main results.

Authors	Study design	Sample size	CGM type	Duration of CGM monitoring	Glycemic outcome	Maternal outcomes	Neonatal outcomes
Majewska [32]	RCT	CGM=50 SMBG=50	Real-time, intermittent	4 weeks	No difference in mean fasting glucose during monitoring between the groups (86.71 vs 85.10, p = 0.437). Lower mean postprandial glycemia in the SMBG group (113.94 vs. 109.52, p=0.011). Similar changes in HbA1c in both groups (p=0.546) Higher frequency of nocturnal hypoglycemia identified with CGM.	No difference in the gestational weight gain, caesarean section (OR 0.84, 95% CI 038-1.87), weeks of gestation at birth (p=0.872) between groups.	A significantly higher incidence of fetal macrosomia in the SMBG group (20% vs. 4.08%, OR 5.62, 95% CI 1.16-27.22). LGA and neonatal hypoglycemia had a higher frequency in the SMBG group, but the differences were not statistically significant (OR 2.38, 95% CI 0.69-8.22, and OR 1.29, 95% CI 0.50-3.28 respectively).
Wei [33]	RCT	CGM early =25 CGM late =30 SMBG=62	Real-time, continuous	4-8 weeks Early: 24-28 weeks Late: 28-36 weeks	Non-significant lower HbA1c in the CGM group (5.5% vs. 5.6%, p =0.089) No significant differences in the mean and standard deviation of the glucose value and MODD were observed between the CGMS subgroups. Insulin was more commonly used in the CGMS group than in the SMBG group (31.3% vs. 12.7%, p=0.02).	A lower proportion of excessive gestational weight gain in the CGM groups than in the SMBG (33.3% vs. 56.4%, p=0.039) No difference in the cesarean section and gestational weeks at birth between groups	No difference in the frequency of macrosomia, LGA, neonatal hypoglycemia and birth weight between groups.
Alfadhli [34]	RCT	CGM=68 SMBG=62	Real-time, continuous	3-7 days	Comparable HbA1c (6.1% vs. 5.7%, p=0.168), mean fasting and postprandial glucose between the two groups at the end of the pregnancy No significant differences in the number of women who required insulin therapy (11.16% vs. 11.4%, p=0.896)	No difference in maternal outcomes between study groups	No difference in the neonatal outcomes between study groups.
Lai [35]	RCT	CGM=77 SMBG=77	Blinded	3 days every 4 weeks	No differences in TIR, mean blood glucose levels and glucose variability parameters between the CGM and SMBG groups	Similar rates of cesarean delivery (54.8% vs. 58.1%, p=0.717) and hypertensive disorders (8.1% vs.	Lower birth weight in the CGM groups than in the SMBG (p=0.015) No difference between CGM and SMBG for macrosomia, LGA,

					Similar HbA1C antepartum in both groups (5.31 vs. 5.35%, p=0.599)	3.2%, p=0.436) in CGM and SMBG groups. The proportion of women within the gestational weight gain recommendations was higher in the CGM group (59.7% vs 40.3%, p=0.046.	preterm birth, hyperbilirubinemia, birth injury, neonatal hypoglycemia, respiratory distress and NICU admission).
Yu [67]	Prospective cohort study	CGM=150 SMBG=190	Retrospective	4 weeks after the diagnosis	CGM reduced glycemic variability and shortened the duration of hyperglycemia or hypo-glycemia compared with baseline (p <0.001). 3.4% of women using CGM experienced hypoglycemia with a duration of >30 minutes/day, compared to 19.4% of the women on SMBG (p <0.001)	CGM use reduced the incidence of preeclampsia (3.4% vs. 10.1%) and cesarean delivery (34.7% vs. 46.6%). MAGE improved with CGM use and was independently associated with preeclampsia risk (OR 3.66; 95% CI 2.16-6.20, p <0.001), but not with cesarean delivery (OR 1.27; 95%CI 0.96-1.68, p=0.101)	
Kestilä [94]	RCT	CGM=36 SMBG=37	Real-time, continuous	47.4±2.5h	Similar HbA1c in both groups (5.4% vs. 5.3%, p=0.15) Antihyperglycemic medication started in a higher proportion CGM than SMBG group (31% vs. 8%, p=0.0149)	No difference in weight gain during pregnancy (12.1 kg vs. 13.9 kg, p=0.26), frequency of cesarean section (22.2% vs. 21.6%, p=0.47), pre-eclampsia or pregnancy-induced hypertension.	No statistically significant differences between the two groups in macrosomia (11.1% vs. 8.1%, p=0.33), neonatal hypoglycemia (13.9% vs. 13.8%, p=0.5), hyperbilirubinemia or NICU transfer (19.4% vs. 30.8%, p=0.11).
Bastobbe [109]	Observational, retrospective, pilot study	CGM=37 SMBG=74	Real-time, intermittent	Form GDM diagnosis until birth	HbA1c levels at birth were higher in CGM pregnancies [5.6% vs. 5.4%, p=0.013].	No difference between groups in hypertensive pregnancy disorders, preterm delivery, and cesarean section.	No difference between groups in weight at birth, the frequency of LGA (6.0% vs. 10.0%, p=0.550), hyperbilirubinemia, neonatal hypoglycemia (6.9% vs. 1.5%, p=0.224) and NICU admission (3.3% vs. 10.0%, p=0.169).
Chen [103]	Prospective, obervational	CGM=57	Blinded	72 hours	Improved identification of hyperglycemia and nocturnal hypoglycemia undetected by self-monitoring.	-	-
Lane [105]	RCT	CGM=23	Blinded (12) Real-time, continuous (11)	4 weeks	No difference in mean sensor glucose level between the blinded and real-time CGM groups (98.9 ± 8.9 mg/dL and 107.5 ± 11.4 mg/dL).	No difference in the frequency of polyhydramnios, cesarean delivery between groups.	No difference in the frequency of macrosomia, LGA, shoulder dystocia, admission to NICU or respiratory distress syndrome between groups.

Paramasivam [97]	RCT	CGM=25<br>SMBG=25	Retrospective	6-day sensor at 28, 32 and 36 weeks' gestation	Mean HbA1c was lower at 37 weeks in CGM than in SMBG (5.2% vs. 5.6%, p <0.006).<br>92% of the CGM group achieved an HbA1c ≤ 5.8% at 37 weeks of gestation compared with 68% of the control group, p=0.012.<br>Similar fasting, pre-meals and 2h post meals glycemia in both groups at 28, 32 and 36 weeks.<br>Numerically, more women in the CGM group received intensive insulin therapy (74% vs. 56%, p=0.195).<br>Neither group experienced severe hypoglycemia.	Maternal weight gain was similar in both groups (10.1 kg vs. 10.3 kg, p=0.917).	No difference in neonatal mortality, fetal anomaly, neonatal hypoglycemia, LGA and NICU admission between groups.
Zhang [107]	RCT	CGM=55<br>SMBG=55	Real-time, intermittent	Not specified	Patients who used CGM had a lower frequency of hypoglycemia (5.45% vs. 21.82%, p=0.012).	A grater rate of weight gain by the end of the pregnancy was observed in the CGM group (90.91% vs. 70.91%, p=0.008).	-
Kwiatkowska [108]	Retrospective	CGM=53<br>SMBG=224	Real-time, intermittent	From insulin therapy initiation until birth	Women in FGM group received insulin treatment earlier (15 vs. 27 weeks; p <0.001) and used it more often (98.1% vs 81.3% times, p=0.005).	Women in the SMGB group had lower pregnancy weight gain (10 vs 12 kg; p=0.033).<br>No significant differences in pregnancy arterial hypertension (9.8% vs 13.1%; p=0.681) and caesarean sections (61.4% vs 64.1%; p=0.898) was observed between groups.	The groups did not differ in birth weight (3243 ± 485 vs 3331 ± 359 g; p=0.206) and frequency of LGA (6.7% vs 5.3%; p=1.0).
Li [42]	Prospective observational	CGM=760	Real-time, continuous	After DGM diagnosis until birth	-	2nd trimester %TIR over 140 mg/dl had acceptable predictive accuracy and specificity for hypertensive disorders of pregnancy (AUROC 0.58) similar to the prediction made using OGTT.	2nd trimester %TIR over 140 mg/dl had acceptable predictive accuracy and specificity for LGA (AUROC 0.58).
Voormolen [111]<br>Rademaker [112]	RCT	CGM=54<br>SMBG=54	Retrospective	After insulin initiation for GDM and <30 weeks of gestation	Similar HbA1c levels between treatment groups.	Reduced risk of preeclampsia with CGM compared with SMBG (RR 0.14; 95% CI 0.02-1.12, p=0.03).<br>No association of TIR, %TAR, %TBR, mean sensor glucose or HbA1c with pre-eclampsia.	No difference in the risk of macrosomia between groups (RR 1.22; 95% CI, 0.55-2.71).<br>No association of TIR, %TAR, %TBR, mean sensor glucose or HbA1c with neonatal hypoglycemia, LGA, or NICU admission.

Valent [136]	RCT	CGM=74<br>SMBG=34	Real-time, continuous	After 20 weeks of gestation until delivery	CGM group had significantly higher %TIR (93 min vs. 88 min at 60–140 mg/dL; p=0.027), lower 24-h and daytime mean glucose and percent time >140 mg/dL compared with SMBG group.	There was no difference in gestational weight gain and hypertensive disorders of pregnancy (26% vs. 17%, p=0.435) between groups.	There was no difference in neonatal weight, LGA, macrosomia, NICU admission, neonatal hypoglycemia and respiratory distress syndrome between groups.
Elkind-Hirsch [116]	RCT	CGM=80<br>SMBG + monthly blinded CGM=40	Real-time, continuous	After GDM diagnosis (8-26 weeks) until delivery	No differences between groups in %TIR 63–140 mg/dl (88.6% vs. 87.4%, p=0.37), TBR, hypoglycemic events, mean glucose or CV were found.<br>More participants with CGM had a <25%TAR than those in SMBG (95.0% vs. 82.5%, p=0.04).	CGM group experienced significantly lower unscheduled caesarean section rates (20.0% vs. 44.4%, p=0.046) and preterm delivery rate (6.8% vs. 18.4%, p=0.041).	Neonates of mothers from CGM group had lower LGA rates (5.0% vs. 18.4%, p=0.019) and NICU admissions (22.5% vs. 44.7%, p=0.013) compared to SMBG group.
Shen [113]	Cohort study	CGM=97	Real-time, intermittent	5-14 days at a mean of 28.8 weeks of gestation	-	-	Each 1-SD increase in maternal nighttime mean glucose level and hours per-day spent in a severe variability glucose mode was associated with 6.0 (95% CI 0.4-11.5) and 6.3 (95% CI 0.4-12.2) percentage points increase in birth weight percentile, respectively.<br>No associations were found between other glucose metrics and birth weight percentile.
Law [114]	Prospective observational study	CGM=162	Blinded	7-day at 30-32 weeks of gestation	-	-	Mean glucose was significantly higher in women who delivered an LGA infant (111.6 mg/dl vs. 104.4 mg/dL, p=0.025).<br>No significant differences in percentage time in, above, or below the target glucose range or in glucose variability measures between those who delivered an LGA or a normal weight infant.
Liang [115]	Prospective cohort study	CGM=1302	Blinded	14-day at a mean gestational age of 26 weeks	-	-	Per 1-SD change in TAR, AUC, nighttime MBG, daytime MBG, and daily MBG was significantly associated with increased risk of any adverse neonatal outcome, with OR: 1.22 (95% CI 1.08–1.36), 1.22 (95% CI 1.09–1.37), 1.18 (95%

CI 1.05–1.32), 1.21 (95% CI 1.07–1.35), and 1.22 (1.09–1.37). Higher level of TIR (per 1-SD, OR 1.30; 95% CI 1.05–1.61), TAR (OR 1.36; 95% CI 1.19–1.56), nighttime MBG (OR 1.54; 95% CI 1.30–1.83), daytime MBG (OR 1.51; 95% CI 1.27–1.79), daily MBG (OR 1.60; 95% CI 1.35–1.91), and MAGE (OR 1.20; 95% CI 1.01–1.42) had a higher risk of LGA. A higher level of TAR was associated with a greater risk of NICU admission (per 1-SD, OR 1.24; 95% CI 1.07–1.44).

RCT= randomized controlled trial; SMBG=self-monitoring of blood glucose; CGM=continuous glucose monitoring; LGA=large for gestational age; NICU=neonatal intensive care unit; TIR=time in range; TAR=time above range; TBR=time below range; CV=coefficient of variation; MBG=mean blood glucose; AUC=area under the curve; OR=odds ratio; RR=relative risk; CI=confidence interval; SD=standard deviation
